# Catatonia Following Oral Ingestion of Cannabis

**DOI:** 10.1192/bjo.2024.682

**Published:** 2024-08-01

**Authors:** Abhinav Tewari, Ashish Pathak, Andrea Pathak

**Affiliations:** 1 Atharv Brain Clinic, Lucknow, India; 2 Essex Partnership University NHS Foundation Trust (EPUT), Basildon, United Kingdom

## Abstract

**Aims:**

Catatonia is a neuropsychiatric syndrome that affects motor, speech, and behavioural functions. The link between catatonia and cannabis use is complex and poorly understood, with limited evidence from case reports about the neuropsychiatric manifestations. This paper aims to describe an unusual presentation of catatonia precipitated by a drink made from cannabis leaves (a.k.a. ‘Bhang’).

**Methods:**

Mr JP, a 22-year-old college student, was admitted to an acute medical ward in North India. The medical team sought psychiatry opinion following the unusual presentation: sudden onset of mutism, staring, and rigidity. Physical examination revealed tachycardia and redness of the eyes. Routine blood investigations, EEG and MRI-Brain were unremarkable. Urine drug screen was positive for cannabis. Initially reluctant due to fear of legal troubles, the accompanying friends later revealed a history of ingestion of cannabis leaves (Bhang) for recreational purposes twelve hours ago. Following the clinical diagnosis of catatonia, the lorazepam challenge test led to improvement in rigidity and verbal responsiveness. No overt psychotic symptoms, such as delusions or hallucinations, were noted at the time or during follow-up. JP had previously experimented with smoked cannabis without any diagnosed psychiatric or medical complications requiring inpatient management. He was abstinent from all forms of cannabis use over the past three months due to college exams and denied any illicit substance use. Over the next two days, as the effects of the ingested cannabis wore off and oral lorazepam (6 mg/day) was continued, JP was back to his previous self with stable vital signs. He was discharged from the hospital with a plan to taper and stop lorazepam on an outpatient basis.

**Results:**

‘Bhang' has been a culturally acceptable cannabis form in the Indian subcontinent for centuries, providing an interesting cultural aspect to the case. This case highlights an unusual clinical instance of cannabis use; Oral ingestion led to a drastic presentation requiring hospitalisation, while the smoked form did not result in any similar sequelae. The study's limitations include the inability to test for synthetic cannabinoids and the lack of objective catatonia scoring scale(s).

**Conclusion:**

With the surging popularity of cannabis use in recent years, it is essential to be aware of its various forms and exercise a high degree of suspicion towards unusual presentations. Further research is needed to understand the link between cannabis use and catatonia at the neurotransmitter level, mediated by individual risk factors.

